# Solution for the Eczema of Dentition

**Published:** 1891-04

**Authors:** 


### Solution for the Eczema of Dentition:

R. Hydrochlorate of cocaine 2 grains.

Bromide of potassium 15 grains.

Pure glycerine } of each ½ ounce. ℳ.

Distilled water }

Rub thoroughly together, and apply to the parts with the soft part of the finger. If insomnia is present, owing to the itching produced by the eruption, a teaspoonful of a syrup made up as follows will be found useful:

R. Bromide of potassium 7 grains.
Syrup of orange 1 ounce. ℳ.

For the cure of the condition, an ointment composed of oxide of zinc, 1 drachm, and vaseline, 3 drachms, may often be employed with advantage.—*Medical News.*

Theine in the Treatment of Neuralgia.—Every now and then cases of neuralgia are reported which have been treated successfully by the hypodermic injection of theine. The local anæsthetic action of this alkaloid was, we believe, first brought to the attention of the profession by Dr. Thomas J. Mays, about four years ago, who, from his experimental and clinical investigation, concluded that its physiological action is not identical with that of caffeine, and that its analgestic action is more prompt and more permanent in neuralgia than that of morphine, or of any of the other agents in common use for the purpose of deadening pain.

